# Consensus-Based Sequential Estimation of Process Parameters via Industrial Wireless Sensor Networks †

**DOI:** 10.3390/s18103338

**Published:** 2018-10-06

**Authors:** Feilong Lin, Wenbai Li, Liyong Yuan

**Affiliations:** 1 Department of Computer Science, Zhejiang Normal University, Jinhua 321004, China; liwenbai@zjnu.edu.cn; 2 Xingzhi College, Zhejiang Normal University, Jinhua 321004, China; yuan@zjnu.edu.cn

**Keywords:** industrial wireless sensor networks, consensus-based sequential estimation, co-design

## Abstract

Process parameter estimation, to a large extent, determines the industrial production quality. However, limited sensors can be deployed in a traditional wired manner, which results in poor process parameter estimation in hostile environments. Industrial wireless sensor networks (IWSNs) are techniques that enrich sampling points by flexible sensor deployment and then purify the target by collaborative signal denoising. In this paper, the process industry scenario is concerned, where the workpiece is transferred on the belt and the parameter estimate is required before entering into the next process stage. To this end, a consensus-based sequential estimation (CSE) framework is proposed which utilizes the co-design of IWSN and parameter state estimation. First, a group-based network deployment strategy, together with a TDMA (Time division multiple access)-based scheduling scheme is provided to track and sample the moving workpiece. Then, by matching to the tailored IWSN, the sequential estimation algorithm, which is based on the consensus-based Kalman estimation, is developed, and the optimal estimator that minimizes the mean-square error (MSE) is derived under the uncertain wireless communications. Finally, a case study on temperature estimation during the hot milling process is provided. The results show that the estimation error can be reduced to less than $3{\;}^{\circ }C$ within a limited time period, although the measurement error can be more than $100{\;}^{\circ }C$ in existing systems with a single-point temperature sensor.

## 1. Introduction

Industrial wireless sensor networks (IWSNs) have been considered to be fundamental technologies that will promote the next industry revolution, such as Industry 4.0 and Industrial Internet. By leveraging the advantages of IWSNs, in terms of flexible deployment and wireless routing, monitoring in hostile environments becomes tractable and the ubiquitous conception on the plant floor becomes feasible, both of which are bottleneck problems in traditional wired monitoring systems. Therefore, more and more efforts have be paid to developing new solutions with IWSNs to improve industrial production. In this paper, we investigate process parameter estimation in a representative industrial scenario where a workpiece is being transferred on the transferring belt. We expect to obtain an accurate estimate of the specific parameter’s value with the aid of IWSN before the workpiece enters into the next process stage.

**Figure 1:**
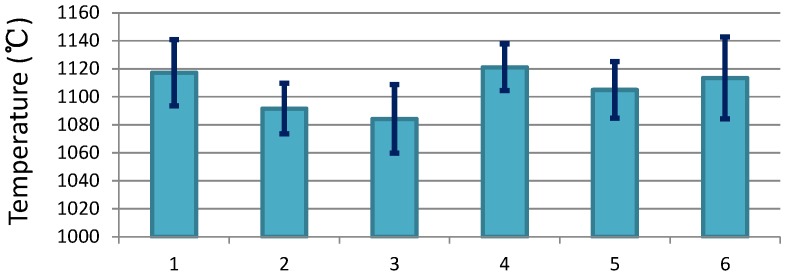
Standard deviation of the temperature sampling error of different steel grades from the rough mill: (1) general structural steel; (2) hull structural steel; (3) high strength low alloy steel; (4) container sheet; (5) carbon structural steel; (6) hot-rolled alloy steel coil. Data were provided by LiuSteel in China.

Process parameter estimation, to a large extent, determines the quality of industrial production [1,2]. However, hostile environments gives parameter samples a serious level of noise.For example, in most existing hot strip milling systems, there is, at most, one temperature sensor equipped at each milling stage. The temperature measurements are polluted in the milling line which is pervaded with vapour and dust. Figure 1 presents the statistical standard deviation of the temperature samples of different steel grades from the rough mill of LiuSteel in China, which shows that the standard deviation can be up to $30{\;}^{\circ }C$. These noisy measurements may lead to faulty milling process control and eventually, result in a low yield of high-quality strip mill. Therefore, precise parameter estimation is demanded in advanced hot milling systems as well as in many other process industries.

IWSN is a promising technique that may improve process parameter estimation. It is capable of enriching sampling points by flexible deployment and decreasing the noise level by collaborative signal denoising. However, the process of determining how to conduct conduct process parameter estimation by IWSN presents two interacting types of challenges. On one hand, IWSN deployment and transmission coordination should adapt to the process industry scenario. Since the workpiece is transferred along the belt, the proper IWSN deployment strategy should be adopted to fulfill the coverage of the region of interest and network connection. In addition, network coordination should be considered with respect to moving target tracking, continuous sampling and information processing, and data exchange with unstable wireless communication in a hostile industrial environment. On the other hand, in the process industry, estimation of the state of the parameter of the moving workpiece is required before it enters into the next process stage. Hence, a local and distributed approach is a more feasible choice than a remote and centralized solution. More importantly, the target estimation algorithm is expected to work well under the IWSN constraints such as network deployment, transmission scheduling, unstable wireless communications, etc.

To address the above issues, a consensus-based sequential estimation (CSE) framework is proposed in this paper. We highlight the novelty and contributions of this work as follows:

- The CSE framework utilizes the co-design of the IWSN deployment and scheduling with the distributed Kalman filter-like parameter state estimator. The relationship and inter-constraints between the IWSN settings and distributed estimation algorithm implementation are investigated.
- In terms of IWSN design, a group-based network deployment strategy together with a TDMA (Time division multiple access)-based scheduling scheme for the network operation is developed. It addresses the sequential monitoring problem as the workpiece moves along the transferring belt. Meanwhile, the vital parameter for state estimation, i.e., iterations of estimation update, is formulated under the constraints of the IWSN settings.
- In terms of the state estimation, the sequential estimation algorithm utilizing the consensus-based Kalman estimation is developed by network design matching, and the optimal estimator minimizing the mean-square error (MSE) is derived under the uncertain wireless communications. The algorithm contributes to the tracking of the time-varying process parameter and reaches estimation consensus within a limited time period.
- Finally, we present a case study by applying CSE to temperature estimation in the hot milling process. The results show that CSE can reduce the estimation error to within $3{\;}^{\circ }C$ before the steel slab enters the rough milling process, although the measurement error can be more than $100{\;}^{\circ }C$ in existing systems with a single-point temperature sensor. In addition, the requirements for IWSN settings are analyzed from an estimation performance perspective, which provides the future research direction of joint optimization of IWSN and process parameter estimation.

The remainder of this paper is organized as follows. A simple review on state-of-the-art IWSN research is presented in Section 2. In Section 3, the problem of industrial process parameter estimation is stated. The sequential monitoring approach, including wireless sensor network design and the coordination scheme, is introduced in Section 4. The CSE algorithm is presented in Section 5. Section 6 presents a case study to evaluate the proposed CSE approach. This paper is finally concluded in Section 7.

## 2. Related Works

In recent years, developed industrial nations have been trying to work towards the next industrial revolution. National plans, e.g., Industry 4.0 by Germany, Industrial Internet by America, Made in China 2025 by China, etc., have been proposed. In all of these new paradigms, the use of industrial wireless sensor networks (IWSNs) is considered to be the fundamental element. IWSNs provide a feasible solution towards realizing ubiquitous monitoring in industrial fields through their flexible deployment and collaborative working manner [3]. Therefore, IWSN has become a research hotspot in recent years.

To deal with coverage and connection problems in different industrial scenarios, various network deployment strategies have been studied. For example, the work in Ref. [4] used group-based IWSN deployment, which divides the objected area into multiple sub-areas, for industrial production line monitoring. Considering the communication requirements under this specific industrial scenario, two group patterns have been derived to represent the inter-/intra- group connections of the group-based IWSN in a simplified way. Another extensively utilized approach is the cluster-based method [5], which adapts to scenarios where multiple agents or actuators exist as cluster heads. Compared to peer-to-peer connections in the group-based IWSN, cluster-based deployment greatly simplifies the network’s topology, while cluster heads have to cope with a much higher workload. Hence, optimization strategies to improve the network efficiency have been studied, such as dynamic cluster formation to balance the network resource distribution of the nodes [6].

Another commonly studied issue is transmission scheduling in IWSN. Generally, the TDMA-based scheduling methodology is utilized since deterministic and predictable data transmission is requested in most industrial applications [7]. The standard protocols for IWSN, e.g., WirelessHART [8], provide time slot allocation-based network transmission scheduling by specified superframe design. Based on the TDMA scheme, various optimization designs with different appealing aspects of performance have been developed. Ref. [9] reviews the latest work on real-time transmission scheduling. The scheduling design for end-to-end reliable data packet transmission is considered in Ref. [10]. Ref. [11] presents a transmission scheduling algorithm for time-bounded data flows, which optimizes the guaranteed time slot (GTS) allocation of IEEE 802.15.4 MAC. Our previous works [12,13] mainly focused on TDMA-based scheduling on multiple radio channels, which aim to descend the transmission delay by improving the spectrum efficiency.

Considering that parameter state estimation in the process industry is the main concern in this work, we simply review the research on Kalman filter-like estimation algorithms or solutions. Olfati-Saber did the pioneering work on the consensus-based distributed Kalman filter [14,15,16]. In Ref. [14], the distributed Kalman-consensus filter (DKCF) algorithm is proposed which has identical observation matrices, requiring the objective to be observable by every sensor. The generalized DKCF with consensus process is introduced in Ref. [15], where non-perfect observation and unstable communication cases are tolerated. In Ref. [16], the optimal design of DKCF, the convergence property, and the responded performance are systematically discussed. Considering the distributed filtering and prediction of time-varying processes, S. Das et al. analyzed the three consensus strategies in DKCF, i.e., innovation consensus in Ref. [17], observations consensus in Ref. [18], and state consensus in Ref. [19]. The results show that state consensus-based DKCF gains a lower MSE than the others. In Ref. [2], parameter state estimation for industrial automation is considered. A distributed collaborative control scheme is proposed based on distributed state estimation. As for the time-varying industrial parameter estimation, Ref. [20] presents a distributed Kalman filter-based state estimation approach which is used to estimate the temperature distribution of steel slab in the study case. We also preliminarily discussed the steel slab temperature estimation during the hot milling process using IWSN in Ref. [21].

However, to service industrial production under the Industry 4.0 paradigm or other similar paradigms, the IWSN design and parameter state estimation generally have to been jointly considered. IWSN deployment is constrained by the process industry scenario; network scheduling is constrained by the movement of the workpiece; and the embedded state estimation algorithms are constrained by the IWSN deployment and scheduling. Although much research on IWSN has been done, there is still a gap in the literature results regarding practical industrial implementations. Based on the existing research, this work focuses on the co-design of IWSN and parameter state estimation in process industries.

## 3. Target Statement

The general target of this work was to construct a process parameter estimation framework using IWSN. In particular, we considered a scenario where the workpiece is moving along the transferring belt and the parameter estimate is required before the workpiece enters into the next process stage. To achieve this general target, two sub-targets, namely the IWSN design and process parameter estimation, need to be considered in a coupled way, which has been seldom focused on before. In the following text, the co-design principles of IWSN and process parameter estimation are stated.

First, IWSN design for process parameter estimation needs to consider adaption to the process industry scenario. In such scenarios, the interested region is long and narrow. Hence, the IWSN design in this work adopts the group-based network deployment strategy, which is predicted to meet the coverage and connectivity requirements. Moreover, due to the movement of workpiece with rigorous timing in the process industry, the TDMA-based scheduling strategy is used to coordinate the network operations, including intra- and inter-group network communication. Detailed network deployment together with the TDMA-based scheduling are presented in Section 4. In addition, in the process industry, parameters of the workpiece are generally time-varying, e.g., the temperature of the steel slab during the milling process. A parameter estimation algorithm is embedded into IWSN to conduct the online estimation.

Second, the process parameter estimation over IWSN needs be adapted to the network topology and scheduling. In this work, the distributed Kalman estimation algorithm is exploited. The Kalman estimation algorithm is capable of tracking time-varying parameters. The distributed approach suits the group-based network topology which provides robust parameter estimation over IWSN. Considering the movement of a workpiece, the estimation algorithm is also constrained by the network design. For example, as the workpiece is transferred along the transferring table, the estimation process has to be shifted along the network groups one by one. Finally, an accurate estimate is expected before the workpiece enters into the next process stage. The details of the process parameter estimation algorithm’s design over IWSN are presented in Section 5.

Intuitively, the IWSN design has a great impact on process parameter estimation, and in contrast, process parameter estimation can also aid in IWSN design optimization, for example, selecting the proper network size and determining the sampling period duration; thus, parameter estimation needs to meet the required precision level. Discussions on these issues are given in the following sections. We also preliminarily demonstrate the relationship between network design and parameter estimation performance in Section 6.

## 4. IWSN Design

**Figure 2:**
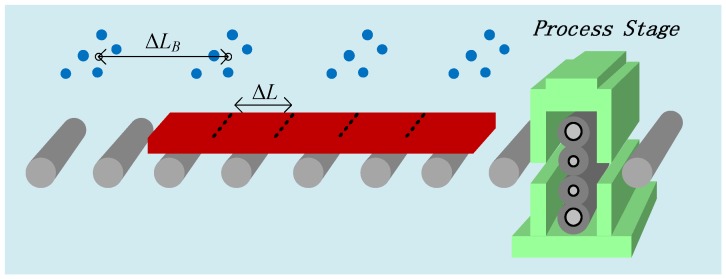
Schematic illustration of the industrial wireless sensor network (IWSN) for process parameter estimation.

Figure 2 presents a sketch map of the process industry, where the workpiece is transferred along the transferring belt towards the next process stage. For IWSN design, some factors of this scenario have to be considered, which are listed in Table 1. As shown in Table 1, the physical size of the workpiece is L×W. If it is relatively long, it can be logically segmented into multiple subregions. Thus, the processing resolution can be increased. The velocity of the workpiece is *v*. The size of the transferring belt is denoted by $L_{B}\times W_{B}$. The IWSN is supposed to monitor the moving workpiece. Hence, the deployment of the sensor network and its coordination of the parameter data sampling, forwarding, exchange, and calculation for process parameter estimation necessitate a dedicated design. This section presents the IWSN design in a sequential way that conducts continuous monitoring of the process parameter.

### 4.1. Group-Based Network Deployment

In this work, the group-based sensor deployment is adopted. As the workpiece moves along the transferring belt, the sensor groups can track and monitor the workpiece in a sequential way from one group to the next. For each period during which the workpiece is covered by a certain sensor group, the sensor can work collaboratively to improve parameter estimation. The design of the group-based IWSN is as follows:

(1) *Sensor*: Suppose that the sensor can be deployed over the transferring belt. It samples process parameters in a non-touch way, e.g., by infrared thermometer. The wireless communication modular embedded into the sensor node supports data exchange with other sensors. The communication radius of all sensors is assumed to be identical, and is denoted by *r*.
(2) *Sensor group*: The sensor group is utilized to cover a relatively small region, for example, one interested subregion of the system. Suppose that each group consists of *N* nodes which are deployed compactly. For one sensor group (*i*), graph $G_{i}\left(V,E\right)$ depicts the topology, where *V* and *E* represent the sets of sensor nodes and their corresponding communicating connections, respectively.
(3) *IWSN construction*: The IWSN in this work was constructed by placing multiple sensor groups along the transferring belt. Note that the transferring belt is narrow, and we assumed that one sensor group could cover the width of the transferring belt. Hence, we were mainly concerned with placing the sensor groups in the transferring direction. The distance between two neighboring groups is defined as the physical distance between the center points of groups $G_{i}$ and $G_{i+1}$, as illustrated in Figure 2. The distance is denoted by $\Delta L_{B}$. Then, along the transferring belt, $M=\lfloor \frac{L_{B}}{\Delta L_{B}}\rfloor$ sensor groups can be deployed, denoted by $G_{i},i=1,2,\cdots ,M$. In addition, to assure the connectivity of the constructed IWSN, it is assumed that $\Delta L_{B}<r$, which can be easily satisfied in practical applications.
(4) *Preliminaries of sequential monitoring*: To conduct the process parameter estimation, some preliminaries are presented. The group-based sensor network is supposed to do the sequential monitoring as the workpiece moves along the transferring belt. The final estimate is required before it enters into the next process stage. Accordingly, the nodes within one group are coupled by a bidirectional communication network in an ad hoc manner and are in charge of collaborative parameter estimation. As for the inter-group communication between each $G_{i}$ and $G_{i+1}$, it is captured by a unidirectional network. The inter-group communication supports $G_{i}$ to deliver the intermediate estimation results to $G_{i+1}$, since the workpiece always moves from one group to the next. The final sensor group ($G_{M}$) obtains the final estimate.

For some scenarios, such as steel strip milling, the workpiece is long and continuously processed. To increase the processing resolution, the workpiece can be logically segmented into multiple subregions with identical lengths of ΔL. We set $\Delta L=\frac{1}{D}\Delta L_{B},\;D\in Z^{+}$, such that one subregion can be handled by one sensor group. For each subregion, $S_{l},l=1,2,\cdots ,\left\lceil \frac{L}{\Delta L}\right\rceil$, dedicated network coordination is needed to fulfill the sequential monitoring and parameter estimation, which is presented in the following text.

### 4.2. Scheduling for Sequential Monitoring

Deterministic operations are generally required for industrial applications. In this work, TDMA-based scheduling is used for network coordination. Recalling Table 1, the workpiece as well as subregion $S_{l}$ are transferred with a given velocity (*v*). During transference, $S_{l}$ passes the sensor groups one by one. The duration is zoomed in from $S_{l}$, changing $G_{i-1}$ to $G_{i}$, and focusing on network scheduling of this duration. Obviously, this schedule can be considered to be the basic one. To fulfill the scheduling of the entire group-based IWSN, one only needs to duplicate this basic schedule. Thus, $S_{l}$ can be sequentially monitored by sensor groups.

**Figure 3:**
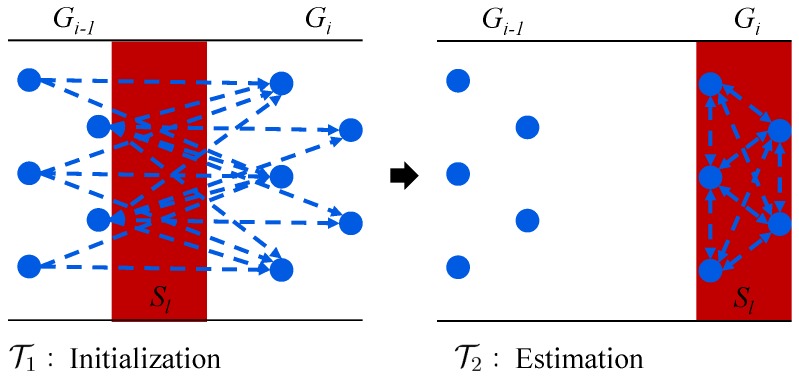
Two phases of the consensus-based estimation.

Further, the sequential monitoring is decomposed into two phases: the initialization phase $T_{1}$ and the estimation phase $T_{2}$, as shown in Figure 3. Assume that if the subregion $S_{l}$ has left the monitoring range of node group $G_{i-1}$, then, the nodes in $G_{i-1}$ terminate their estimation process ($T_{2}$) and transmit the local estimates to the sensor nodes in $G_{i}$. After receiving the estimates from $G_{i-1}$, the nodes in $G_{i}$ aggregate these data and trigger their initialization phases ($T_{1}$) to set the aggregated data as a priori knowledge. When subregion $S_{l}$ is detected by $G_{i}$, the estimation phase ($T_{2}$) is triggered. During the estimation phase, $T_{2}$, local estimates of $G_{i}$ are executed to update the priori knowledge by assimilating the new measurements and aggregating the neighboring data. If the time period is long enough before $S_{l}$ leaves $G_{i}$, multiple local estimation iterations can be embedded. When $S_{l}$ has left $G_{i}$, the nodes in $G_{i}$ first terminate the estimation phase ($T_{2}$) and then forward the estimates to the next group ($G_{i+1}$) for the next round of parameter estimation of subregion $S_{l}$.

The sensor network needs a dedicated scheduling process to enforce the interwinded examination of the initialization phase ($T_{1}$) and the estimation phase ($T_{2}$) between neighboring groups. As the industrial process control requires rigorous and deterministic information transmission, the TDMA-based frame, which is widely used for industrial applications, e.g., WirelessHART and ISA100.11a, is employed for transmission scheduling. In the TDMA-based frame, time is divided into slots of equal size, and each slot can be assigned to one sensor node to transfer one data packet to another node. It is assumed that one data packet can accommodate the local estimate at each sensor node.

Corresponding to the aforementioned phases, $T_{1}$ and $T_{2}$, the schedule can be divided into two segments with time lengths of $T_{1}$ and $T_{2}$. $T_{1}$ is the time from when $S_{l}$ leaves $G_{i-1}$ to when $S_{l}$ arrives at $G_{i}$. $T_{2}$ is the subsequent time until $S_{l}$ leaves $G_{i}$. They can be obtained by $T_{1}=\frac{\Delta L_{B}-\Delta L}{v}$ and $T_{2}=\frac{\Delta L}{v}$. Then, the dedicated slot allocation of the schedule follows. At the beginning of segment $T_{1}$, $T_{\mathrm{ini}}$ slots are reserved for the forwarding of local estimates from $G_{i-1}$ to $G_{i}$ as the initial state of the estimation process in $G_{i}$. As the workpiece moves along the transferring belt, when subregion $S_{l}$ arrives at group $G_{i}$, segment $T_{2}$ begins. Within $T_{2}$, a couple of rounds of parameter estimation can be accommodated. At each round, the nodes in $G_{i}$ sample parameter $S_{l}$ within $T_{\mathrm{sam}}$ slots. Then, they exchange the data within $T_{\mathrm{exc}}$ slots. In the following $T_{\mathrm{cal}}$ slots, the estimation algorithm for this round is executed by combining the local estimates from both $G_{i-1}$ and $G_{i}$, and their new measurements. Hence, the sampling period of sensors can be set to $T_{s}\geq T_{\mathrm{sam}}+T_{\mathrm{exc}}+T_{\mathrm{cal}}$. One way to improve the estimation performance is to decrease the sampling period, and thus, to increase the number of iterations of state estimation. In the following section, the determination of the scheduling frame with the purpose of minimizing $T_{s}$ is presented.

### 4.3. Determination of the Scheduling Frame

**Figure 4:**
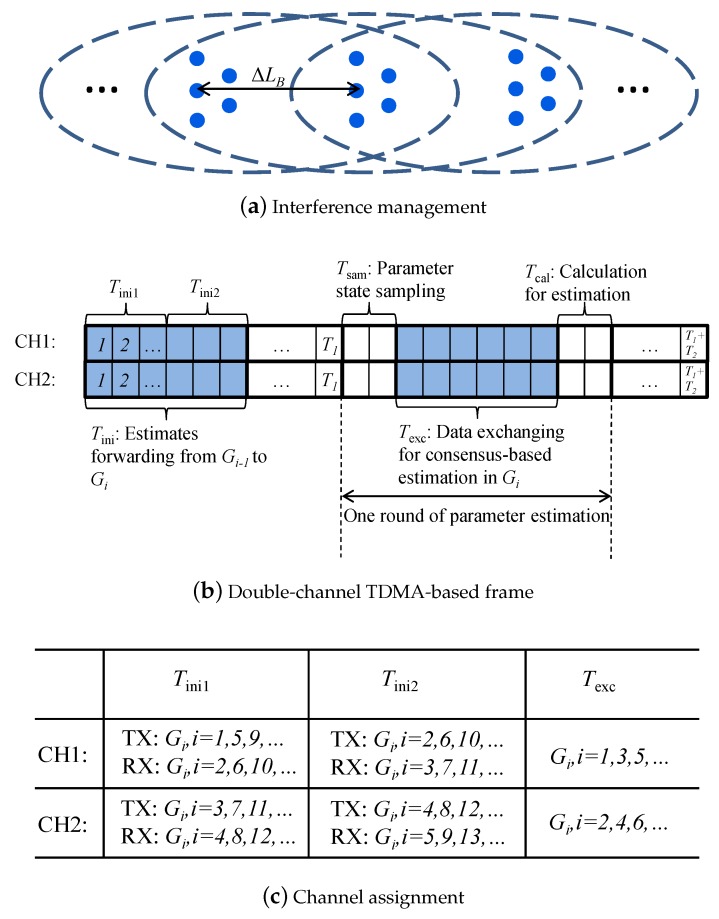
Illustration of TDMA-based scheduling for consensus-based sequential estimation (CSE).

Suppose that the sensor is equipped with a half-duplex transceiver and its power is controllable. Intuitively, minimizing the interference range can improve the network scheduling. For the group-based network deployment in this work, the optimum choice of radio power is used to exactly guarantee the interconnection between two neighboring groups, thus meeting the requirement of $\Delta L_{B}<r<2\Delta L_{B}$ as shown in Figure 4a. In the following text, the use of the double-channel TDMA-based frame to implement the network scheduling is implemented.

As shown in Figure 4b, within $T_{1}$, the inter-group communication needs to be scheduled. Note that $G_{i}$ can simultaneously receive the radio signals from $G_{i-1}$ and $G_{i+1}$ when they respectively forward estimates to $G_{i}$ and $G_{i+2}$. To address this hidden terminal problem, the frequency division method was used in this work. As illustrated in Figure 4c, this involves the assignment of two orthogonal channels, CH1 and CH2. In $T_{\mathrm{ini}1}$, sensor groups $\left\{G_{i},i=1,5,9,\cdots \right\}$ forward estimates to sensor groups $\left\{G_{i},i=2,6,10,\cdots \right\}$ on channel CH1, and sensor groups $\left\{G_{i},i=3,7,11,\cdots \right\}$ forward estimates to sensor groups $\left\{G_{i},i=4,8,12,\cdots \right\}$ on channel CH2. Then, in $T_{\mathrm{ini}2}$, sensor groups $\left\{G_{i},i=2,6,10,\cdots \right\}$ forward estimates to sensor groups $\left\{G_{i},i=3,7,11,\cdots \right\}$ on channel CH1, and sensor groups $\left\{G_{i},i=4,8,12,\cdots \right\}$ forward estimates to sensor groups $\left\{G_{i},i=5,9,13,\cdots \right\}$ on channel CH2. As for the intra-group communication within $T_{2}$, sensor groups $\left\{G_{i},i=1,3,5,\cdots \right\}$ operate on channel CH1 and sensor groups $\left\{G_{i},i=2,4,6,\cdots \right\}$ operate on channel CH2. Thus, the interference can be avoided.

To implement the proposed scheme, there are some parameters that remain to be determined in order to enforce sequential monitoring of the workpiece, namely, $T_{\mathrm{rec}}$, $T_{\mathrm{sam}}$, $T_{\mathrm{exc}}$, and $T_{\mathrm{cal}}$. $T_{\mathrm{sam}}$ and $T_{\mathrm{cal}}$ are related to the hardware of the sensor node, which can be set according to the capability of the hardware. In the following text, we determine $T_{\mathrm{rec}}$ and $T_{\mathrm{exc}}$. Consider that within one group, sensors are close to each other. In accordance with the node-based scheduling approach presented in Ref. [22], for a frame to schedule the transmission of *N* mutually interfered nodes, *N* slots are needed. Hence, in our studied network, we set $T_{\mathrm{ini}1}=T_{\mathrm{ini}2}=N$ and $T_{\mathrm{exc}}=N$.

Based on the above design of the network and operation schedule, it is expected that process parameter estimation of the subregions will be obtainedwithin the limited running time, i.e., $T=\frac{L_{B}}{v}$ with finite *M* sensor groups. The following remark highlights the results that facilitate process parameter estimation.

**Remark** **1.** *With the double-channel TDMA-based frame, the minimum sampling period can be set to*

(1) $$T_{s}=T_{\mathrm{sam}}+T_{\mathrm{cal}}+N.$$

*Thus, during $T_{2}$,*

(2) $$N_{\mathrm{ite}}=\left\lfloor \frac{T_{2}}{T_{s}}\right\rfloor$$

*samples can be conducted by each sensor. As the workpiece is transferred along the belt, a total of*

(3) $$N_{\mathrm{tot}}=MN_{\mathrm{ite}}$$

*iterations of estimation updates can be executed.*

In this work, the process parameter estimation is addressed by a consensus-based sequential estimation method, which is presented in the following section.

## 5. Consensus-Based Sequential Estimation

As the process parameter generally varies over time, it can be formulated as follows:

(4) $$\dot{x}\left(t\right)=A\left(t\right)x\left(t\right)+u\left(t\right),$$

where x(t) is the H-dimensional parameter state; A(t) is the state transition matrix of the process parameter; u(t) is the exterior input or influence from the exterior. By adopting IWSN, one sensor node (*i*) samples the process parameter at time instant *t*; then, the noisy measurement can be expressed as

(5) $$y_{i}\left(t\right)=C_{i}x\left(t\right)+\upsilon_{i}\left(t\right),$$

where $C_{i}$ is the H×H observation matrix, and $\upsilon_{i}\left(t\right)$ is the zero-mean measurement noise with covariance $R_{i}$. It is supposed that the sampling noises satisfies $E\left[\upsilon_{i}\left(t_{1}\right)\upsilon_{j}^{T}\left(t_{2}\right)\right]=R_{i}\delta_{ij}\delta_{t_{1}t_{2}}$, where $\delta_{ij}$ is the unit impulse function.

Alongside the network deployment and the corresponding scheduling in the previous section, we present the CSE algorithm to finish the final estimation of the parameter state of the workpiece. In this section, the consensus-based Kalman estimation is first introduced, and the unscented transformation dealing with the nonlinear process prediction is then presented. As CSE is operated within one subregion, the superscript *l* for the subregion index is omitted for convenience in the following section.

### 5.1. Consensus-Based Kalman Estimation

For group-based network deployment, node *i* in sensor group $G_{i^{\prime }}$ is considered, where *i* and $i^{\prime }$ are independent. ${\hat{x}}_{i}\left(k\right)$ and ${\tilde{x}}_{i}\left(k\right)$ denote the estimate and prior estimate (or prediction) of the state x(k) by node *i* at the *k*th sampling period. In particular, k=0 represents the moment of the beginning of $T_{1}$, and $k=1,2,\cdots ,N_{\mathrm{ite}}$ represent sampling moments from the beginning of $T_{2}$. Based on the settings, the distributed Kalman filter [15] is utilized to implement the CSE in the *k*th sample period (strictly, for $k=1,2,\cdots ,N_{\mathrm{ite}}$, x(k) should be written as $x(T_{1}+\left(k-1\right)T_{s})$. For simplicity, x(k) is used), which can be formed from the following equations.

*Initialization*:

(6) $$\begin{array}{c} {\hat{x}}_{i}\left(0\right)=\frac{1}{|M_{i}\left(0\right)|}\sum_{j\in M_{i}\left(0\right)\subseteq G_{i^{\prime }-1}}{\hat{x}}_{j}\left(0\right), \end{array}$$

(7) $$\begin{array}{c} {\tilde{x}}_{i}\left(1\right)=A_{i}\left(0\right){\hat{x}}_{i}\left(0\right), \end{array}$$

*Consensus-based Kalman estimation*:

(8) $${\hat{x}}_{i}\left(k\right)={\tilde{x}}_{i}\left(k\right)+K_{i}\left(k\right)\left(y_{i}\left(k\right)-C_{i}{\tilde{x}}_{i}\left(k\right)\right)+\sum_{j\in N_{i}\left(k\right)\subseteq G_{i^{\prime }}}\gamma_{ij}\left({\tilde{x}}_{j}\left(k\right)-{\tilde{x}}_{i}\left(k\right)\right),$$

*Prediction*:

(9) $${\tilde{x}}_{i}\left(k+1\right)=A_{i}\left(k\right){\hat{x}}_{i}\left(k\right),$$

for $k=1,2,\cdots ,N_{\mathrm{ite}}$, where $K_{i}\left(k\right)$ is the Kalman filter gain; $\left\{\gamma_{ij}\right\}$ are the weight coefficients of the consensus-based estimation; and $M_{i}\left(0\right)\subseteq G_{i^{\prime }-1}$ and $N_{i}\left(k\right)\subseteq G_{i^{\prime }}$ represent the neighbor sets of node *i* for data forwarding and data exchange at the corresponding time period, respectively. Both neighbor sets $M_{i}\left(0\right)$ and $N_{i}\left(k\right)$ are time-dependent and are decided by network topology and real-time wireless communication. Regarding the group-based network deployment presented in Section 4.1, it was supposed that $|M_{i}\left(0\right)|>0$ and $|N_{i}\left(k\right)|\geq 0$.

Following the network operation schedule, as shown in Figure 4b, the initialization phase, consisting of Equations (6) and (7), is executed with $T_{1}$ only once, where Equation (6) refers to the reception and fusion of thte estimates from the previous sensor group, and Equation (6) refers to the prediction during $T_{1}$. The consensus-based Kalman estimation phase, Equation (8), and the prediction phase, Equation (9), are executed $N_{\mathrm{ite}}$ times during $T_{2}$. At a system level, due to the constraints to the movement of the workpiece, $MN_{\mathrm{ite}}$ updates can be conducted for the consensus-based Kalman estimation.

In the following text, the optimal gain matrix, $K_{i}\left(k\right)$, for this consensus-based Kalman filter is determined. The introduced errors are denoted at each step of the consensus-based sequential estimation algorithm by ${\tilde{e}}_{i}\left(k\right)={\tilde{x}}_{i}\left(k\right)-x\left(k\right)$, and ${\hat{e}}_{i}\left(k\right)={\hat{x}}_{i}\left(k\right)-x\left(k\right)$. Further, the error covariance matrices ${\tilde{P}}_{ij}\left(k\right)=E\left[{\tilde{e}}_{i}\left(k\right){\tilde{e}}_{j}^{T}\left(k\right)\right]$ and ${\hat{P}}_{ij}\left(k\right)=E\left[{\hat{e}}_{i}\left(k\right){\hat{e}}_{j}^{T}\left(k\right)\right]$ are defined. ${\tilde{P}}_{i}\left(k\right)$ and ${\hat{P}}_{i}\left(k\right)$ are short for ${\tilde{P}}_{ii}\left(k\right)$ and ${\hat{P}}_{ii}\left(k\right)$, respectively. In this context, the purpose of this work is to find the optimal $K_{i}\left(k\right)$ that minimizes the mean-square error (MSE) of the estimation, and which can be found by the following theorem.

**Theorem** **1.** *Regarding the consensus-based Kalman filter in Equation (8), the optimal $K_{i}\left(k\right)$ that minimizes the MSE of the estimation is*

(10) $$K_{i}\left(k\right)=\left({\tilde{P}}_{i}\left(k\right)+{\tilde{P}}_{N_{i}}\left(k\right)\right)C_{i}^{T}{\left(C_{i}{\tilde{P}}_{i}\left(k\right)C_{i}^{T}+R_{i}\right)}^{-1},$$

*where*

(11) $${\tilde{P}}_{N_{i}}\left(k\right)=\sum_{j\in N_{i}\left(k\right)}\gamma_{ij}\left({\tilde{P}}_{ij}\left(k\right)-{\tilde{P}}_{i}\left(k\right)\right).$$

**Proof.**  Using the definitions of the introduced errors by each step of the consensus-based sequential estimation algorithm, the following equation is obtained:

(12) $${\hat{e}}_{i}\left(k\right)=W_{i}\left(k\right){\tilde{e}}_{i}\left(k\right)+K_{i}\left(k\right)\upsilon_{i}\left(k\right)+\sum_{j\in N_{i}\left(k\right)}\gamma_{ij}\left({\tilde{e}}_{j}\left(k\right)-{\tilde{e}}_{i}\left(k\right)\right),$$

where $W_{i}\left(k\right)=I-K_{i}\left(k\right)C_{i}$. Then, the error covariance after the consensus-based estimation obtained from

(13) $${\hat{P}}_{i}\left(k\right)=W_{i}\left(k\right){\tilde{P}}_{i}\left(k\right)W_{i}^{T}\left(k\right)+W_{i}\left(k\right){\tilde{P}}_{N_{i}}\left(k\right)+{\tilde{P}}_{N_{i}}^{T}\left(k\right)W_{i}^{T}\left(k\right)+{\tilde{D}}_{i}\left(k\right)+K_{i}\left(k\right)R_{i}K_{i}^{T}\left(k\right),$$

where ${\tilde{P}}_{N_{i}}\left(k\right)$ is defined by Equation (11), and

(14) $${\tilde{D}}_{i}\left(k\right)=\sum_{r\in N_{i}\left(k\right)}\sum_{s\in N_{i}\left(k\right)}\gamma_{ir}\gamma_{si}\left({\tilde{P}}_{rs}\left(k\right)-{\tilde{P}}_{ri}\left(k\right)-{\tilde{P}}_{is}\left(k\right)+{\tilde{P}}_{i}\left(k\right)\right).$$

Note that for any random variable ($x\in R^{n}$) with covariance *P*, the following relation holds:

$$E\left[x^{T}x\right]={∥E\left[x\right]∥}^{2}+\mathrm{tr}\left[P\right],$$

where tr[·] is the trace of a matrix. Then, solution $K_{i}\left(k\right)$ which minimizes the MSE of the estimation is equivalent to

(15) $$\underset{K_{i}\left(k\right)}{\mathrm{minimize}}\;\mathrm{tr}\left[{\hat{P}}_{i}\left(k\right)\right].$$

Consequently, the necessary condition for the optimal $K_{i}\left(k\right)$ satisfies

(16) $$\frac{\partial \mathrm{tr}[{\hat{P}}_{i}\left(k\right)]}{\partial K_{i}\left(k\right)}=-2\left(I-K_{i}\left(k\right)C_{i}\right){\tilde{P}}_{i}\left(k\right)C_{i}^{T}-2C_{i}{\tilde{P}}_{N_{i}}\left(k\right)+2K_{i}\left(k\right)R_{i}=0.$$

Through sorting, the optimal $K_{i}\left(k\right)$ by Equation (10) can be obtained. The proof has been finished. □

Note that, during the industrial process, many parameters are nonlinear. Hence, the prediction of the state and error covariance cannot be linearly updated. To address this, unscented transformation is utilized for the nonlinear process prediction, which is presented in the following subsection.

### 5.2. Unscented Transformation for Nonlinear Process Prediction

The unscented Kalman filter (UKF) uses the principle that a set of discretely sampled points can be used to parameterise the mean and covariance; the estimator for nonlinear systems yields an equivalent performance to the KF for linear systems [23]. First, it generates a set of (2H+1) sigma points ${\left\{{\hat{X}}_{i}^{r}\left(k\right)\right\}}_{r=0}^{2H}$ with a mean of ${\hat{x}}_{i}\left(k\right)$ and a covariance of ${\hat{P}}_{i}\left(k\right)$ as follows

(17) $$\begin{array}{c} {\hat{X}}_{i}^{0}\left(k\right)={\hat{x}}_{i}\left(k\right),\\ W_{i}^{0}=\kappa /\left(H+\kappa \right)\\ {\hat{X}}_{i}^{r}\left(k\right)={\hat{x}}_{i}\left(k\right)+{\left\{\sqrt{\left(H+\kappa \right){\hat{P}}_{i}\left(k\right)}\right\}}_{r},\\ W_{i}^{r}=1/\left(2\left(H+\kappa \right)\right),\;r=1,2,\cdots ,H,\\ {\hat{X}}_{i}^{r}\left(k\right)={\hat{x}}_{i}\left(k\right)-{\left\{\sqrt{\left(H+\kappa \right){\hat{P}}_{i}\left(k\right)}\right\}}_{r-H},\\ W_{i}^{r}=1/\left(2\left(H+\kappa \right)\right),\;r=n+1,\cdots ,2H, \end{array}$$

where κ∈R and the term ${\left\{\sqrt{\left(H+\kappa \right){\hat{P}}_{i}\left(k\right)}\right\}}_{r}$ represents the *r*th column of the square root of matrix $\sqrt{\left(H+\kappa \right){\hat{P}}_{i}\left(k\right)}$. Then, these sigma points are propagated through the state function to generate the predicted points by

(18) $${\tilde{X}}_{i}^{r}\left(k+1\right)=A_{i}\left(k\right){\hat{X}}_{i}^{r}\left(k\right)+u_{i}\left(k\right)$$

for r=0,1,⋯,2H.

Finally, the predicted state uses the weighted average of the predicted points as

(19) $${\tilde{X}}_{i}\left(k+1\right)=\sum_{r=0}^{2H}W_{i}^{r}{\tilde{X}}_{i}^{r}\left(k+1\right),$$

and the corresponding error covariance can be calculated by

(20) $${\tilde{P}}_{ij}\left(k+1\right)=\sum_{r=0}^{2H}W_{i}^{r}\left({\tilde{X}}_{i}^{r}\left(k+1\right)-{\tilde{X}}_{i}\left(k+1\right)\right)\times {\left({\tilde{X}}_{j}^{r}\left(k+1\right)-{\tilde{X}}_{j}\left(k+1\right)\right)}^{T}.$$

Through integration with the initialization of Equations (6) and (7), the state update Equations (8), (10), and (13), and the state prediction Equations (19) and (20), the consensus-based sequential estimation can be performed. The specific algorithm for the implementation of process parameter estimation can be organized by Algorithm 1, where the operation of sensor *i* in group $G_{i^{\prime }}$ under the control of the tailored schedule is shown as the example.

### 5.3. Computational and Communication Complexities

In CSE, the computational cost step is the update of error covariance matrices (${\tilde{P}}_{i}$) and cross error covariance matrices (${\tilde{P}}_{ij}$) when computing the Kalman filtering gain matrix by Equation (10). Hence, the computational complexity is $O(H^{2})$, where H is the dimension of the state error vector ($e_{i}$).

The communication complexity is lower, since in our TDMA-based transmission scheduling method, each sensor node transmits each data packet twice in one estimation round. First, sensor *i* transmits the estimate $\left[{\tilde{x}}_{i}\left(k\right),{\tilde{e}}_{i}\left(k\right)\right]$ to the neighbors within the incumbent sensor group for consensus-based estimation. Second, sensor *i* transmits the predicted estimate $\left[{\tilde{x}}_{i}\left(k+1\right),{\tilde{e}}_{i}\left(k+1\right)\right]$ to the neighbors in the succeeded sensor group to provide the initial state.

**Algorithm 1** CSE Algorithm
 1:// **In duration $T_{1}$ of the schedule**: 2:Receive state estimates $\left\{x_{j}\left(0\right),j\in M_{i}\left(0\right)\subseteq G_{i^{\prime }-1}\right\}$ and obtain the aggregated estimate, ${\hat{x}}_{i}\left(0\right)$, by Equation (6); 3:Obtain the updated estimate, ${\tilde{x}}_{i}\left(1\right)$, by Equation (7); 4:// **In duration $T_{2}$ of the schedule**: 5:k←1; 6:
**while** $k\leq N_{\mathrm{ite}}$ **do**
 7:  Acquire the new sample $y_{i}\left(k\right)$; 8:  Exchange the prior estimate ${\tilde{x}}_{i}\left(k\right)$ with neighbors $N_{i}\left(k\right)\subseteq G_{i^{\prime }}$; 9:  // Consensus-based Kalman estimation with five steps:10:  **Step 1:** Calculate the Kalman gain, $K_{i}\left(k\right)$, by Equation (10);11:  **Step 2:** Obtain the estimate, ${\hat{x}}_{i}\left(k\right)$, by Equation (8);12:  **Step 3:** Obtain the error covariance of estimation ${\hat{P}}_{i}\left(k\right)$ by Equation (13);13:  **Step 4:** Predict state ${\tilde{X}}_{i}\left(k+1\right)$ by the unscented transformation consisting of Equations (17)–(19);14:  **Step 5:** Predict the error covariance of estimation ${\tilde{P}}_{ij}\left(k+1\right)$ by (20);15:  k←k+1;16:
**end while**



## 6. Case Study

In this work, we validated the proposed CSE approach through a simulation study of steel slab temperature estimation during the hot strip milling process. In particular, we focused on the inter-stand section between the reheating furnace and rough milling, as shown in Figure 2. We tried to monitor the temperature of the steel slab by IWSN as it is transferred on the belt, and then, we expected to derive an accurate estimate of the temperature distribution before the steel slab entered the rough mill.

### 6.1. Case Description

During the hot milling process, the steel slab has a flat shape. The temperature variation is mainly caused by heat conduction and loss in the thickness direction [24]. This case study only considered the temperature variation along the thickness direction, and logically cut the slab into H slices. Let $x={\left[x_{1},\cdots ,x_{H}\right]}^{T}$, which represents the temperature distribution of the steel slab at different thickness layers. Then, the temperature state transition can be reorganized by

(21) x(t+1)=A(t)x(t)−u(t),

where

(22) $$A\left(t\right)=\left[\begin{array}{cccc} 1-\alpha_{1} & \alpha_{1} & & 0\\ \alpha_{2} & 1-2\alpha_{2} & \alpha_{2} & \\ & \ddots & \ddots & \\ & \alpha_{H-1} & 1-2\alpha_{H-1} & \alpha_{H-1}\\ 0 & & \alpha_{H} & 1-\alpha_{H} \end{array}\right]$$

with $\alpha_{h}=\frac{\Delta t\lambda_{x}}{\Delta h^{2}\rho_{x}c_{x}}$, h=1,⋯,H, and

(23) $$u\left(t\right)=\left[\begin{array}{c} \frac{\Delta t\sigma \varepsilon }{\Delta h\rho_{x}c_{x}}\left({x_{1}}^{4}-x_{\infty }^{4}\right)\\ 0_{(H-2)\times 1}\\ \frac{\Delta t\sigma \varepsilon }{\Delta h\rho_{x}c_{x}}\left({x_{H}}^{4}-x_{\infty }^{4}\right) \end{array}\right].$$

For the detailed derivation of the discrete model of heat conduction of the steel slab during hot strip milling, please refer to our previous work [20]. The formulation of the noisy measurement is based on Equation (5) in Section 5.

Note that only the top surface of the slab can be sampled, and consider that the temperature distribution of the slab is symmetric regarding the thickness direction. Thus, the observation matrix only has two non-zero elements, $C_{i}\left(1,1\right)$ and $C_{i}\left(H,H\right)$, regarding the measurement of the slab temperature. In the following text, the wireless sensor network that was used to implement the temperature sampling is introduced.

In accordance with the 2050 mm hot strip milling system, the transferring belt length between the reheating furnace and rough mill was 48 m. The physical size of the steel slab was 9 m × 1.5 m × 0.3 m. The moving speed of the slab was 3 m/s. The initial temperature out from the reheating furnace was $1200{\;}^{\circ }C$. The related thermal properties of the carbon steel are listed in Table 2, where *x* denotes the temperature state. Note that different steel grades may have slightly differentiated thermal properties, but the proposed CSE for their temperature estimation is standard.

To conduct the sequential monitoring of the slab, it was first segmented into 6 subregions along the rolling direction, and each subregion was discretized into 30 layers along the thickness direction. The studied IWSN consisted of 32 groups, and each group involved 5 sensors. Thus, each subregion could be sampled by 5 nodes in one sampling period. The sample noise was normally distributed with zero mean and a variance of R=100. The sampling period was set to 0.5 s. The scheduling strategy in Section 4 was used to determine the schedule for communication and consensus-based estimation. The time length of the slot was set to 10 ms in accordance with standard industrial wireless protocols.

Simulations of this work were conducted on the NS3 network simulator which has been extensively applied to verify works on wireless sensor networks. The results are presented in the following text.

### 6.2. Sequential Temperature Monitoring Results

**Figure 5:**
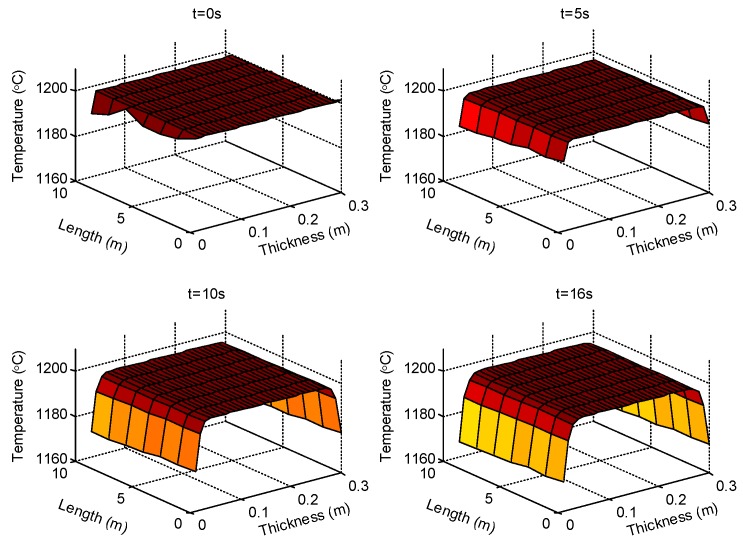
The estimated temperature distribution of the slab at different times.

We start by presenting the visualized results of the slab temperature distribution during the transferring process. Figure 5 captures the temperature distribution estimations of the steel slab at different time instants. The transfer of the slab along the section between the reheating furnace and the rough mill consumes about 16 s. During the transfer, the slab continuously loses heat to the air which results in gradual temperature descending. The subfigures in Figure 5 show the temperature descending process of the slab estimated by CSE. From the beginning, the furnace sends the slab to the transferring belt with a even temperature distribution at $1200{\;}^{\circ }C$. As the slab is transferred, the temperature of the layers on the surface descend, but the temperature of the layers inside the slab (about covering the thickness of 20 cm) is almost not affected by the surface heat lost within the time used for intersection transferring. In the final distribution, the temperature of surface descends to about $1173{\;}^{\circ }C$. Note that subregions of the slab have slightly differentiated temperature distributions due to the different arrival times at the rough mill. The gap of the surface temperature between the first subregion and the last one is about $3{\;}^{\circ }C$.

**Figure 6:**
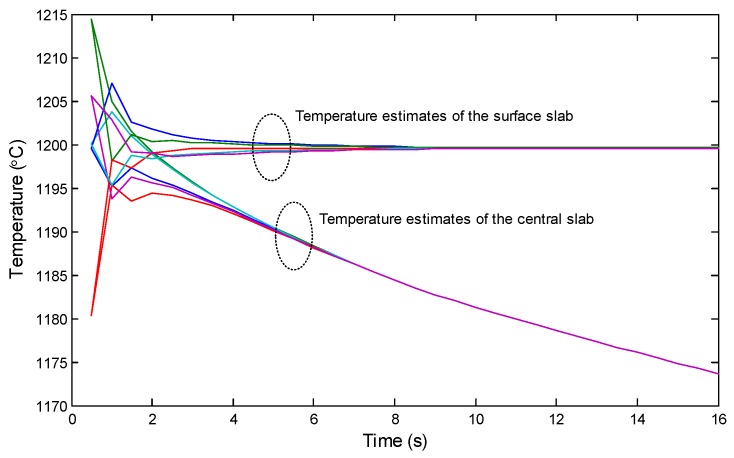
The surface and central temperature estimates by different nodes during transfer of the slab into the interstand section.

Figure 6 shows the process of CSE for the slab by the designed network. For clear presentation, only the surface and central temperature estimates are shown. The top cluster of the curves shows the estimates of the surface of the slab by each group of nodes when the slab is transferred along the belt, and correspondingly, the bottom cluster of the curves shows the estimates of the center of the slab. The results show that with the proposed CSE, the estimates by different nodes can quickly converge, although the sampling process of the sensor nodes contains noise. In the case shown in the figure, the estimation error of the surface of the slab by each node remains below $1{\;}^{\circ }C$ after 5 s, and the estimation error of the center of the slab by each node remains below $1{\;}^{\circ }C$ after 8 s. The difference in convergence times is caused by the innovation part of the consensus-based Kalman filter in CSE. Specifically, on the surface, the innovation update benefits from both the new sample and the consensus calculation. However, inside the slab without sampling, the innovation update can only obtained by the consensus calculation. As a result, the convergence speed of the estimation inside the slab is relatively slow.

**Figure 7:**
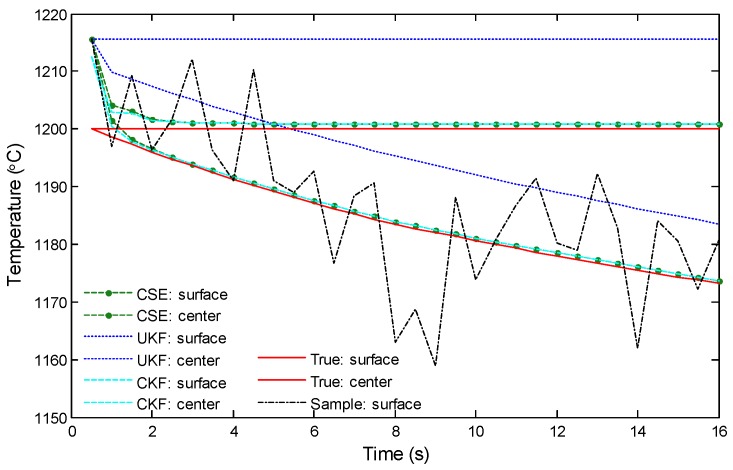
Comparison of the estimated temperatures by CSE, the centralized Kalman Filter (CKF), and the unscented Kalman filter (UKF).

In the following text, the comparison among the proposed CSE, the centralized Kalman Filter (noted by CKF) based estimation, and the unscented Kalman filter (noted by UKF) based estimation are presented. In CKF, we assumed that one central sensor node conducted the estimation by integrating the samples from all of the neighbors. In UKF, we assumed that the central node conducted the estimation independently. Figure 7 presents the comparison results, where the true values of the temperature variation and the samples are also shown in the figure. The sequential results were obtained from one group of the sensors in the network. The figure shows that with CSE, the temperature state of the slab can be well estimated from the seriously noised samples. The estimation performance of CSE is very close to the centralized approach CKF. Estimations by both CSE and CKF quickly approximate the true value. However, without consensus-based estimation, the pure UKF does not approximate to the true value within the running time. Specifically, for the surface layer, the estimation cannot approach the true value due to limited sampling time for UKF-based estimation, and for central layers, the estimation is greatly affected by the initial value since there is no consensus-based calculation and sampling inside the slab. Similar to the data produced from the practical production line, as illustrated in Figure 1, the result shows that single point-based parameter estimation is susceptible to the effects of environmental noise. The comparison results obviously demonstrate the effectiveness of CSE.

**Figure 8:**
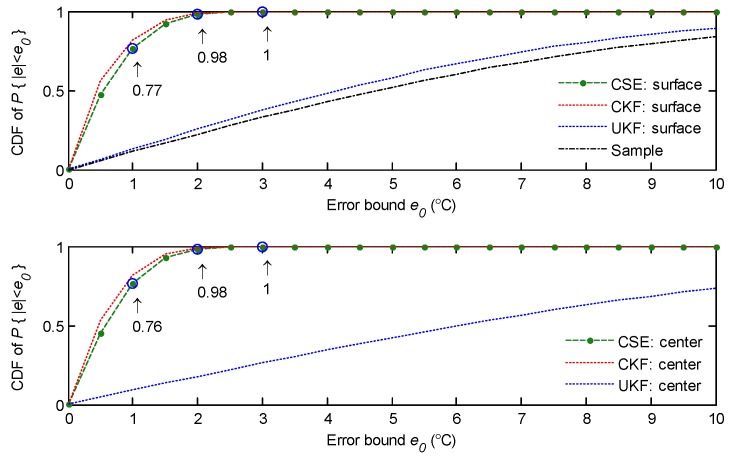
The CDF of the absolute estimation error.

The above results give the intuitional temperature distribution by one snapshot. In the following text, the cumulative distribution function (CDF) of the absolute estimation error (denoted by |e|) is analysed by Monte Carlo simulations. The results of the CFD after 100,000 simulations are presented in Figure 8. The first subfigure presents the CDF of the absolute estimation error for the surface of the slab. It shows that, with CSE, the CDF of the absolute estimation error, $P\{|e|\leq 1{\;}^{\circ }C\}=0.76$, $P\{|e|\leq 2{\;}^{\circ }C\}=0.98$, and $P\{|e|\leq 3{\;}^{\circ }C\}$ are approximately 1. Moreover, the statistical results show that CSE has a similar performance to CKF in the CDF of estimation error. This implies that the distributed approach is as good as the centralized approach in our simulated scenario. The results demonstrate that CSE can greatly improve the estimation performance within a limited sampling time period, and also gains much better estimation performance than the UKF-based estimation. For the estimation of the center of the slab, as the CDF in the second subfigure in Figure 8 shows, CSE has as good of a performance as the estimation of the surface of the slab. However, the UKF-based estimation includes a worse estimation error CDF for the center of the slab than that of the surface of the slab, since the central layer cannot be sampled.

### 6.3. Estimation of Robustness with Uncertain Transmission

In an industrial plant, wireless communication is influenced by the time-varying channel conditions which results in the packet loss. It is observed that the packet loss would damage the sequential temperature estimation, as the packet loss breaks off the state update of the Kalman filter.

**Figure 9:**
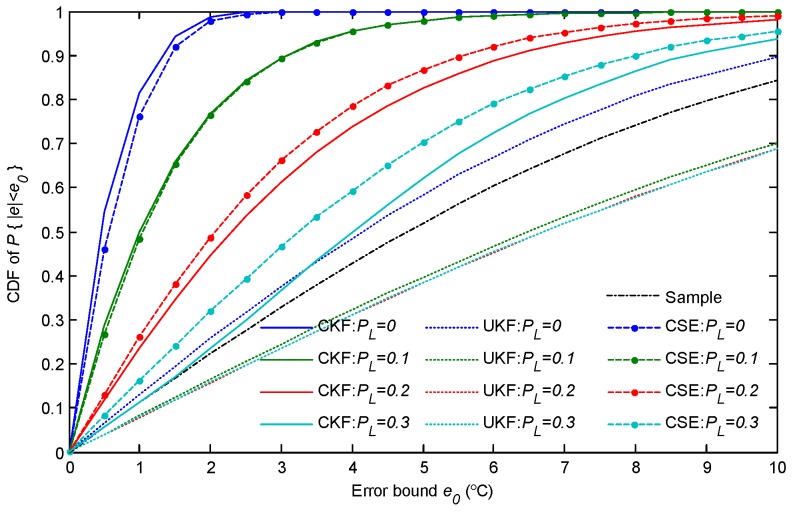
The cumulative distribution function (CDF) of the absolute error of surface temperature estimation with different packet loss probabilities.

This subsection evaluates the estimation performance with the uncertain wireless transmission. Several interferences were deployed among the network which interfered with the packet transmission with a probability of $P_{L}$. The CDFs of the estimation error were counted under different packet loss conditions, and the results for surface temperature estimation are shown in Figure 9. It shows that the temperature estimation performance is severely decreased by the packet loss. For CSE, when the packet transmission is lost with a probability of $P_{L}=0.1$, the probability is $P\{|e|\leq 3{\;}^{\circ }C\}=0.9$, and the maximum estimation error can be up to $8{\;}^{\circ }C$. As the packet loss probability ($P_{L}$) increases, the estimation performance worsens quickly. Unexpectedly, the centralized approach, CKF, has a worse performance than CSE when the packet loss probability is larger than 0.1. The results show that, in the cases of $P_{L}=0.2$ and $P_{L}=0.3$, the estimation accuracy is lower than CSE and the the maximum estimation error can exceed $10{\;}^{\circ }C$. This is caused by the single point weakness of the centralized approach in uncertain wireless communications, which just proves the robustness of CSE in parameter estimation. For UKF, the estimation error can be even larger than the sampling noise when $P_{L}>0.1$. It is caused by the frequent interruptions during the state update when filtering. Once the previous temperature state of the slab cannot be delivered, the temperatures, especially the ones of central layers, are missed. As a result, the new estimated temperature will be much lower than the state of the true temperature. In this sense, the wireless network enhances the estimation robustness of CSE by providing a consensus-based state exchange.

**Figure 10:**
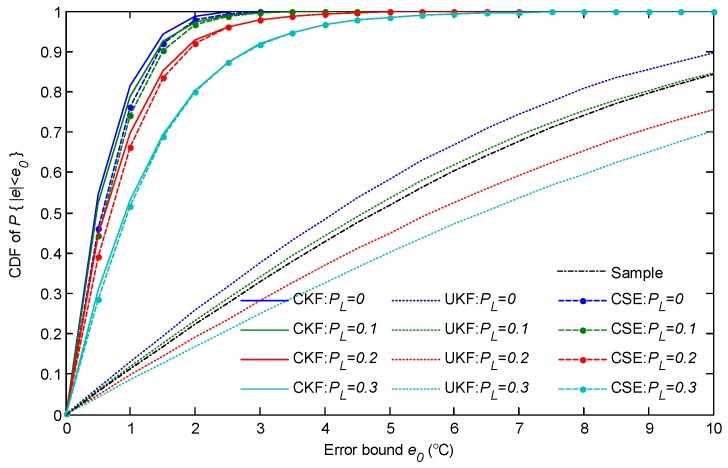
The CDF of the absolute error of surface temperature estimation with different packet loss probabilities; one retransmission was scheduled for each packet transmission.

To reduce the influence of the packet loss on estimation, the retransmission was considered as one effective strategy. In the following simulations, each packet was scheduled with one retransmission chance. The results are presented in Figure 10. They show that with the help of retransmission, both of estimation performances by CSE and CKF improved, since the retransmission increases the probability of successful packet transmission. The CKF slightly outperforms CSE since the synthetical packet loss probability is lower than 0.1 with retransmission, even when $P_{L}=0.3$. However, as UKF needs to guarantee multi-hop but single-link transmission performance during sequential monitoring, its estimation performance is still not good enough, although one retransmission is scheduled.

The estimation performance of the central layer also deteriorates as pack loss occurs. Similarly, the retransmission can effectively relieve the performance deterioration. The detailed results are omitted here.

### 6.4. Evaluation with Difference Network Sizes

Intuitively, when the network comprises more sensor nodes, this brings more samples and makes the estimation results more accurate. However, the presence of more sensor nodes also brings greater challenges, such as deployment cost and network scheduling, especially in the process industry. This subsection evaluates the temperature estimation performance with different networks deployments. The network deployments were adjusted by parameter *K*, which was used in the group-based deployment strategy in Section 4.1. Then, the network consisting of 16 groups and the network of consisting of 8 groups were added to the simulations. Correspondingly, the intergroup distances were set to 3 m and 6 m, and the sampling periods were set to 1 s and 2 s, respectively.

**Figure 11:**
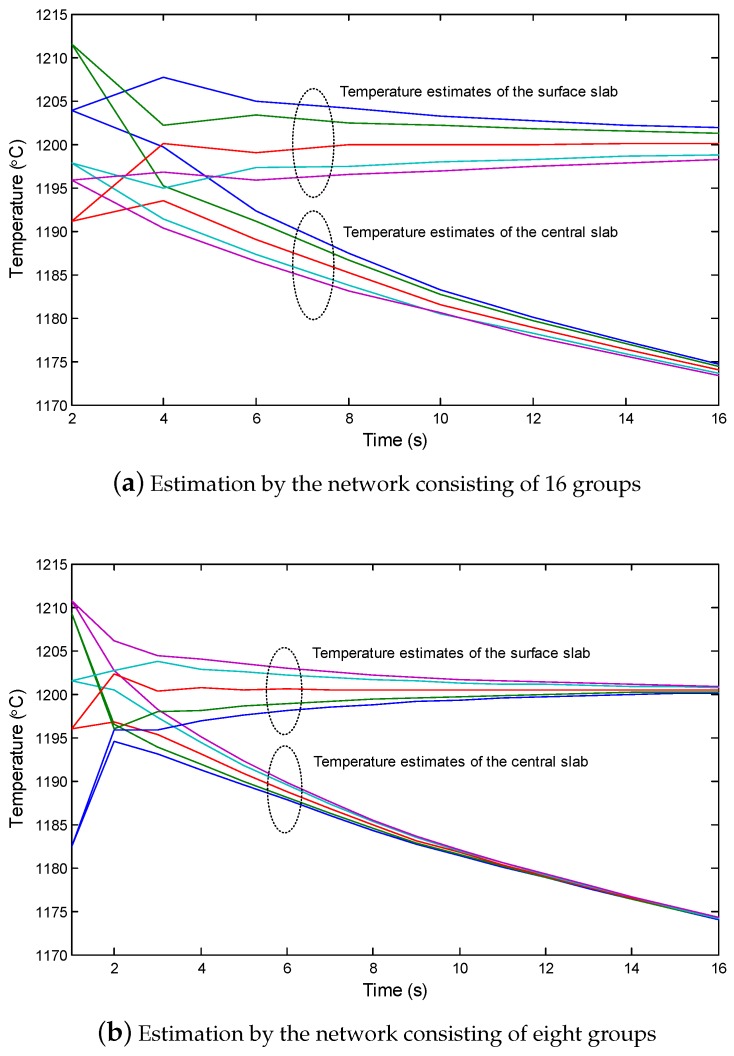
The surface and central temperature estimates during the slab transfer into the interstand section.

Figure 11a,b shows the surface and central temperature estimates during the transfer of the slab in the interstand section, respectively. When the network groups reduced, the convergence of the temperature estimation was slow. For example, when the number of groups was 8, the estimates of nodes, even at the final time period, was still largely different from each other.

**Figure 12:**
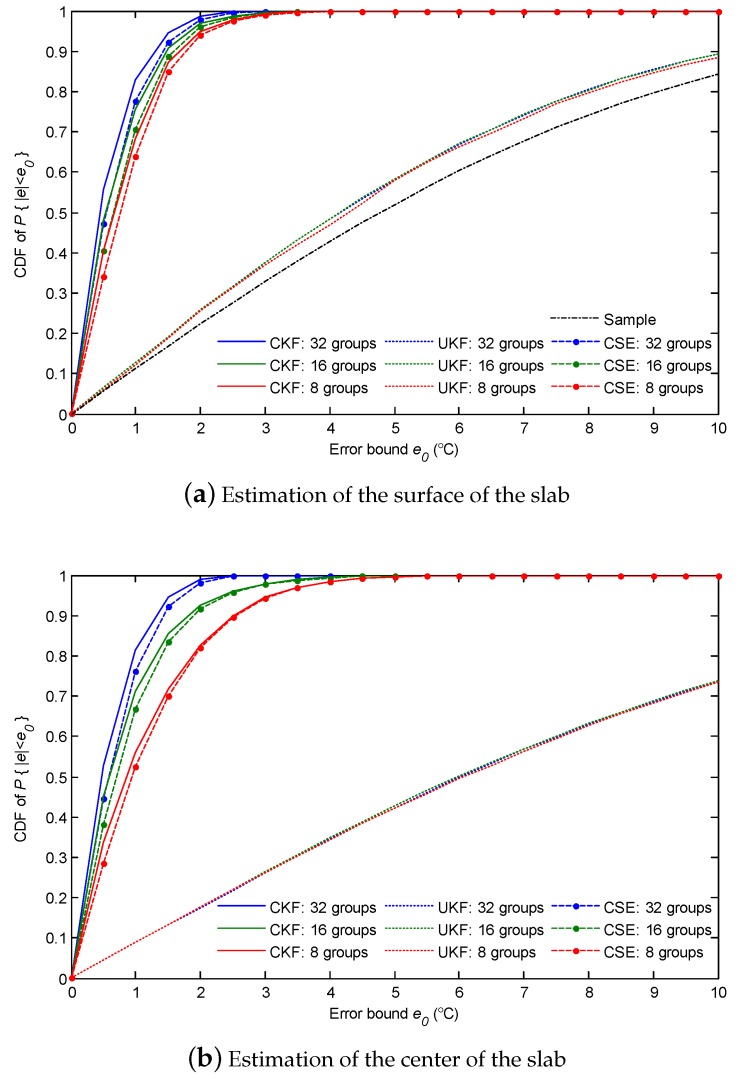
The CDF of the absolute error of temperature estimation with different network sizes.

Further, the CDFs were also counted by Monte Carlo simulations, and the results are presented in Figure 12. The statistical results show that the slimming of the network leads to a loss of estimation performance. For the estimation error of the surface of the slab, as shown in Figure 12a, the CDF of CSE is slightly worsened by reducing the network groups. However, the CSE still performs better than the single point UKF. Positively, CSE obtains similar CDFs to CKF for different network sizes. As shown in Figure 12b, the CDF of the estimation error inside the slab has a salient deterioration as the network groups are reduced. The results indicate that the different network sizes have diverse estimation performances. One can optimize the network design according to the tolerable temperature estimation error.

## 7. Conclusions

This paper presented the state estimation of a process parameter using IWSN. We argued that IWSN design and parameter state estimation are deeply coupled in process industry scenarios. Considering this, a co-design framework, named consensus-based sequential estimation (CSE), was proposed. Under the CSE framework, the group-based network deployment together with the TDMA-based scheduling strategy was adopted. Then, by network design matching, the CSE algorithm utilizing the consensus-based Kalman estimation was developed, and the optimal estimator was derived to minimize the mean-square error (MSE) under uncertain wireless communications. In addition, the requirements for IWSN design from an estimation performance perspective were analyzed which indicated the need for joint optimization of IWSN and process parameter estimation as a future research direction.

## Figures and Tables

**Table 1:** Factors of the process industry scenario related to IWSN design.

Notation	Factors Related to the Process Industry Environment
*L*	Length of the workpiece along the transferring direction
*W*	Width of the workpiece
*v*	Transferring velocity of the workpiece
$L_{B}$	Length of the transferring belt
$W_{B}$	Width of the transferring belt

**Table 2:** Thermal properties of the carbon steels used in this study [24].

Property	Value
$\rho [\mathrm{kg}/m^{3}]$	$7843.76-0.2958\left(x-273\right)-5.65\times 10^{-5}{\left(x-273\right)}^{2}$
λ[W/mK]	$20.14+9.313\times 10^{-3}\left(x-273\right)$
c[J/kgK]	$4.583x-4720.3+1.109\times 10^{9}x^{-2},$ x∈[800,1000)
	12.476x−11501, x∈[1000,1042)
	−32x+34871.2, x∈[1042,1060)
	$5.987x-10068.18+5.21\times 10^{9}x^{-2},$ x∈[1060,1184)
	0.15x+429.85, x∈[1184,1665)
ε	$\frac{x-273}{1000}\left[0.125\frac{x-273}{1000}-0.38\right]$+1.1
$\sigma [W/m^{2}K^{4}]$	$5.67\times 10^{-8}$

## References

[1] Åkerberg J., Gidlund M., Björkman M. Future research challenges in wireless sensor and actuator networks targeting industrial automation. Proceedings of the 2011 9th IEEE International Conference on Industrial Informatics.

[2] Chen J., Cao X., Cheng P., Xiao Y., Sun Y. (2010). Distributed collaborative control for industrial automation with wireless sensor and actuator networks. IEEE Trans. Ind. Electron..

[3] Kumar Somappa A.A., Øvsthus K., Kristensen L.M. (2014). An industrial perspective on wireless sensor networks—A survey of requirements, protocols, and challenges. IEEE Commun. Surv. Tutor..

[4] Lin C., Deng D., Chen Z., Chen K. (2016). Key design of driving industry 4.0: Joint energy-efficient deployment and scheduling in group-based industrial wireless sensor networks. IEEE Commun. Mag..

[5] Cenedese A., Luvisotto M., Michieletto G. (2017). Distributed clustering strategies in industrial wireless sensor networks. IEEE Trans. Ind. Inf..

[6] Gholami M., Brennan R.W. (2016). A Comparison of Alternative Distributed Dynamic Cluster Formation Techniques for Industrial Wireless Sensor Networks. Sensors.

[7] Dujovne D., Watteyne T., Vilajosana X., Thubert P. (2014). 6TiSCH: deterministic IP-enabled industrial internet (of things). IEEE Commun. Mag..

[8] International Electrotechnical Commission (2010). 62591: Industrial Communication Networks—Wireless Communication Network and Communication Profiles–WirelessHART.

[9] Lu C., Saifullah A., Li B., Sha M., Gonzalez H., Gunatilaka D., Wu C., Nie L., Chen Y. (2016). Real-time wireless sensor-actuator networks for industrial cyber-physical systems. Proc. IEEE.

[10] Dobslaw F., Zhang T., Gidlund M. (2016). End-to-end reliability-aware scheduling for wireless sensor networks. IEEE Trans. Ind. Inf..

[11] Hanzalek Z., Jurcik P. (2010). Energy efficient scheduling for cluster-tree wireless sensor networks with time-bounded data flows: Application to IEEE 802.15. 4/ZigBee. IEEE Trans. Ind. Inf..

[12] Lin F., Chen C., He T., Ma K., Guan X. (2017). A separation principle for resource allocation in industrial wireless sensor networks. Wirel. Netw..

[13] Lin F., Chen C., Xu Q., Hua C., Guan X. (2016). A separate design principle for priority-aware packet collection in industrial cyber-physical systems. EURASIP J. Wirel. Commun. Netw..

[14] Olfati-Saber R. Distributed Kalman filter with embedded consensus filters. Proceedings of the 44th IEEE Conference on Decision and Control.

[15] Olfati-Saber R. Distributed Kalman filtering for sensor networks. Proceedings of the 46th IEEE Conference on Decision and Control.

[16] Olfati-Saber R. Kalman-consensus filter: Optimality, stability, and performance. Proceedings of the 48th IEEE Conference on Decision and Control.

[17] Das S., Moura J.M.F. Distributed state estimation in multi-agent networks. Proceedings of the 38th IEEE International Conference on Acoustics, Speech and Signal Processing.

[18] Das S., Moura J.M.F. (2015). Distributed Kalman Filtering With Dynamic Observations Consensus. IEEE Trans. Signal Process..

[19] Das S., Moura J.M.F. (2017). Consensus + Innovations Distributed Kalman Filter With Optimized Gains. IEEE Trans. Signal Process..

[20] Chen C., Yan J., Lu N., Wang Y., Yang X., Guan X. (2015). Ubiquitous Monitoring for Industrial Cyber-Physical Systems Over Relay-Assisted Wireless Sensor Networks. IEEE Trans. Emerg. Top. Comput..

[21] Lin F., Zhu S., Chen C., Guan X. Process Parameter Estimation Oriented Industrial Wireless Sensor Networks: A Sequential Approach. Proceedings of the IEEE International Conference on Communications (ICC).

[22] Ergen S.C., Varaiya P. (2010). TDMA scheduling algorithms for wireless sensor networks. Wirel. Netw..

[23] Julier S., Uhlmann J., Durrantwhyte H.F. (2000). A new method for nonlinear transformation of means and covariances in filters and estimates. IEEE Trans. Autom. Control.

[24] Zhou S.X. (2003). An integrated model for hot rolling of steel strips. J. Mater. Process. Technol..

